# Immunomodulatory Functions of TNF-Related Apoptosis-Inducing Ligand in Type 1 Diabetes

**DOI:** 10.3390/cells13201676

**Published:** 2024-10-10

**Authors:** Marton Fogarasi, Simona Dima

**Affiliations:** 1 Center of Excellence in Translational Medicine, Fundeni Clinical Institute, 022328 Bucharest, Romania; 2 Faculty of Medicine, Carol Davila University of Medicine and Pharmacy, 050474 Bucharest, Romania

**Keywords:** type 1 diabetes, TRAIL, apoptosis, autoimmunity, immunoregulation, T cells

## Abstract

Tumor necrosis factor (TNF)-related apoptosis-inducing ligand (TRAIL) is a member of the TNF protein superfamily and was initially identified as a protein capable of inducing apoptosis in cancer cells. In addition, TRAIL can promote pro-survival and proliferation signaling in various cell types. Subsequent studies have demonstrated that TRAIL plays several important roles in immunoregulation, immunosuppression, and immune effector functions. Type 1 diabetes (T1D) is an autoimmune disease characterized by hyperglycemia due to the loss of insulin-producing β-cells, primarily driven by T-cell-mediated pancreatic islet inflammation. Various genetic, epigenetic, and environmental factors, in conjunction with the immune system, contribute to the initiation, development, and progression of T1D. Recent reports have highlighted TRAIL as an important immunomodulatory molecule with protective effects on pancreatic islets. Experimental data suggest that TRAIL protects against T1D by reducing the proliferation of diabetogenic T cells and pancreatic islet inflammation and restoring normoglycemia in animal models. In this review, we aimed to summarize the consequences of TRAIL action in T1D, focusing on and discussing its signaling mechanisms, role in the immune system, and protective effects in T1D.

## 1. Introduction

Tumor necrosis factor (TNF)-related apoptosis-inducing ligand (TRAIL/Apo2L/CD253) was initially known as a pro-apoptotic molecule that is expressed on the surface of various cell types, including T cells, dendritic cells, natural killer (NK) cells, macrophages, and natural killer T (NKT) cells, depending on their activation status [1]. It induces apoptosis in cancer cells, infected cells, transformed cells, and primary immune cells by interacting with its cognate receptors [2,3]. Compared to other TNF superfamily members, such as CD95L, TRAIL can selectively induce apoptosis in tumor cells without causing cytotoxic effects in healthy cells. On the other hand, TRAIL has the ability to initiate pro-survival signaling, contributing to tumor progression, cell differentiation [4], invasion, and metastasis [5,6]. TRAIL has many important functions in regulating the immune system, including immunomodulatory functions that help maintain immune homeostasis [7].

Type 1 diabetes (T1D) is a multifactorial disease that occurs as a consequence of chronic inflammation of the pancreatic islets [8]. It is characterized by immune cell infiltration, ultimately leading to the destruction of insulin-producing pancreatic β-cells [9]. Although T1D involves multiple factors in the initiation of β cell destruction, it is primarily mediated by autoimmune T cells. Experimental and clinical studies have shown that TRAIL is involved in the development and progression of autoimmunity. It has been suggested that TRAIL exerts a protective effect on the development and progression of T1D through several mechanisms. The association of TRAIL with T1D has been demonstrated in many studies involving both animal models and human patients. Thus, systemic delivery of the adenoviral TRAIL gene in mice showed an anti-inflammatory effect on pancreatic islets [10]. Conversely, in TRAIL-deficient mice or when TRAIL was blocked by injecting a soluble receptor, the mice developed characteristics of T1D with increased autoimmune inflammation of pancreatic islets [11]. Low levels of TRAIL have been measured in patients with T1D at disease onset, which increased once treatment was initiated [12]. Collectively, these studies suggest that TRAIL prevents the development of autoimmune inflammation in pancreatic islets.

## 2. Structure of TRAIL and Its Receptors

The TRAIL molecule belongs to the TNF-ligand superfamily and is expressed as a membrane-bound protein on immune cells such as NK cells [13,14], cytotoxic T cells [15], monocytes and macrophages [16,17,18], dendritic cells [19], and neutrophils [20,21]. The biological functions of TRAIL are complex, as it is involved not only in the regulation of the apoptotic process but also in promoting pro-survival signaling, development, and cell proliferation [22].

TRAIL can be found anchored at the cell surface and is proteolytically shed to generate a soluble fragment [23]. Structurally, it is a glycoprotein with type II transmembrane topology, composed of an N-terminal cytoplasmic tail, a transmembrane domain, a stalk region, and an extracellular C-terminal domain [24]. The TRAIL monomeric protein shows 65% homology between humans (281 amino acids) and mice (291 amino acids). Functionally, TRAIL assembles into a homotrimeric structure and binds to three receptor molecules [25,26] to be biologically active. To fulfill its biological functions, TRAIL interacts with four receptors on the cell surface, DR4/TRAIL-R1, DR5/TRAIL-R2, DcR1/TRAIL-R3, and DcR2/TRAIL-R4 [27,28,29,30], and can bind to the soluble receptor osteoprotegerin [31]. TRAIL’s functions vary depending on the receptor it binds to. Interacting with DR4 and DR5 receptors induces apoptotic or pro-survival signaling in cells, whereas DcR1 and DcR2 are decoy receptors that inhibit TRAIL-induced apoptosis due to the absence of a functional cytoplasmic death domain [32,33,34,35]. DcR1 is bound to the cell surface and lacks the intracellular death domain, while DcR2 is a transmembrane protein with a nonfunctional death domain. Mechanistically, DcR1 outcompetes TRAIL, thereby interfering with DR5-associated DISC assembly, while DcR2 and DR5 interact with DISC components and block caspase activation, thus inhibiting TRAIL-induced apoptosis [32,33]. Osteoprotegerin blocks osteoclastic differentiation and bone resorption [36,37].

## 3. TRAIL-Induced Signaling Pathways

TRAIL has a complex biological function and is capable of triggering various cellular responses. In addition to inducing the classical pro-apoptotic pathway, TRAIL is also engaged in activating pro-survival signaling.

### 3.1. Pro-Apoptotic TRAIL Signaling Pathway

DR4 and DR5 receptors are characterized by the presence of extracellular cysteine-rich domains, a transmembrane region, and an intracellular death domain, which play a crucial role in transducing apoptotic signaling [38,39]. TRAIL binding to these receptors induces a conformational change in both the extracellular structure of the receptor and the intracellular death domain. These rearrangements facilitate the interaction of the TRAIL–receptor complex with the Fas-associated death domain (FADD), which initiates the recruitment of procaspase-8 or -10 via their death domains (DD). This interaction leads to the formation of the death-inducing signaling complex (DISC) [38,40,41,42]. As a result, caspase-8 is activated and released as an active enzyme into the cytoplasm, where it activates caspase-3, -6, and -7. These activated caspases are then poised to execute the apoptotic death program [40,43].

#### 3.1.1. Intrinsic and Extrinsic Pathways of Apoptosis

**Figure 1:**
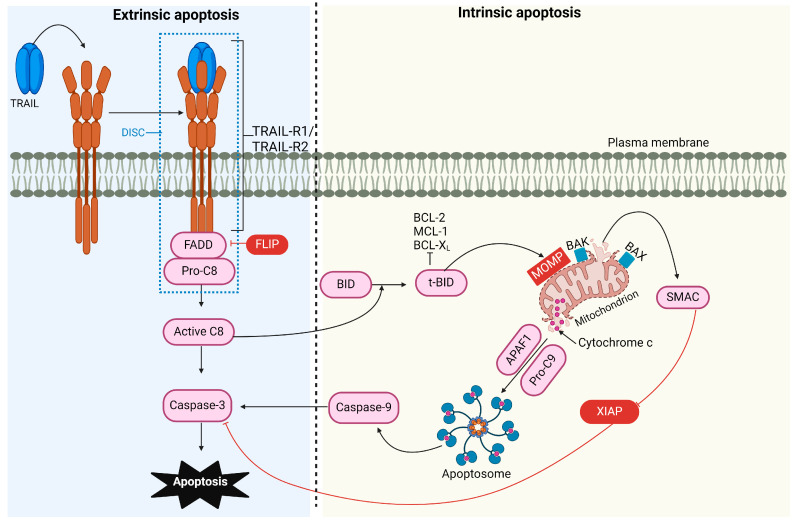
TRAIL-induced pro-apoptotic signaling pathway. The binding of TRAIL to TRAIL receptor 1 (TRAIL-R1) and/or TRAIL-R2 recruits FADD and caspase-8, forming the DISC. The FLICE-like inhibitory protein (FLIP) can compete with caspase-8 for FADD binding. The activation of caspase-8 is enhanced through cullin 3 ubiquitylation and, in type I cells, can activate caspase-3, leading to apoptosis. In type II cells, the processing of effector caspases is inhibited by the X-linked inhibitor of apoptosis protein (XIAP), necessitating additional signaling for apoptosis to occur. Caspase-8 cleaves the BH3-interacting domain death agonist (BID), which translocates to the mitochondria, activating BCL-2 antagonist killer 1 (BAK) and BCL-2-associated X protein (BAX). This causes mitochondrial outer membrane permeabilization (MOMP), leading to the release of SMAC (mitochondria-derived activator of caspase) and cytochrome C. SMAC can block XIAP’s inhibitory effect, while cytochrome C, together with apoptotic protease activating factor 1 (APAF-1), mediates the assembly of the apoptosome, which activates caspase-9 and amplifies caspase-3 activity.

Apoptosis, or programmed cell death, is a complex process essential for regulating physiological cell growth and maintaining tissue homeostasis. This process occurs through two distinct pathways: the intrinsic (or mitochondrial) pathway and the extrinsic (or receptor-mediated) pathway [44] (Figure 1).

##### The Intrinsic Apoptotic Pathway

The B-cell lymphoma-2 (BCL-2) protein family regulates the intrinsic pathway and involves signaling from intracellular pro-apoptotic molecules released by the mitochondria [45]. Under cellular stress and apoptotic stimuli conditions, the BCL-2 protein family is activated, causing mitochondrial outer membrane permeabilization (MOMP). As a result, proteins normally localized in the mitochondrial intermembrane space, such as cytochrome C, apoptosis-inducing factor (AIF) [46], endonuclease G [47], second mitochondria-derived activator of caspase/direct inhibitor of apoptosis-binding protein with low pI (SMAC/DIABLO), and serine protease high-temperature requirement 2 (HtrA2)/Omi [48], are released into the cytosol. Intrinsic apoptosis can proceed through two mechanisms: caspase-dependent and caspase-independent pathways. In the caspase-dependent pathway, cytochrome C, apoptotic protease activating factor 1 (APAF-1), and dATP interact to assemble a multiprotein complex called the apoptosome. This structure activates caspase-9, which activates caspase-3, initiating proteolysis and ultimately leading to cell apoptosis. SMAC/DIABLO and HtrA2 indirectly contribute to caspase activation by inactivating caspase inhibitors known as inhibitors of apoptosis proteins (IAPs) [49]. In the caspase-independent pathway [50], AIF and endonuclease G [47] translocate to the nucleus, initiating chromatin condensation and DNA fragmentation.

##### The Extrinsic Apoptotic Pathway

In the extrinsic pathway (or receptor-mediated pathway), signaling is transmitted via extracellular protein ligands that interact with their cognate death receptors (DRs) expressed on the cell surface. This pathway plays a crucial role in maintaining homeostasis during immune development and in pathological conditions. Apoptosis is essential during lymphocyte differentiation, ensuring that functional antigen receptors are selected and maintained while cells with receptors that recognize self-antigens are eliminated [51,52,53,54]. Dysregulation of this pathway can lead to pathological conditions such as autoimmunity, cancer, immune deficiency, and neurodegenerative disorders. In this pathway, damaged cells are removed through the interaction of the TNF-ligand family with death receptors on unhealthy cells [55]. The binding of ligands to their cognate receptors leads to the assembly of the DISC, which initiates the activation of caspase-8 and -10. These activated proteases subsequently activate effector caspases, culminating in cell apoptosis [56].

Engagement of the intrinsic or extrinsic apoptotic pathway depends on the cell type and is suggested to be determined by the amount of active caspase-8 generated at the DISC [57]. In type I cells, such as thymocytes and T cells, sufficient levels of caspase-8 are generated at the DISC to induce apoptosis via caspase-3 and -7 signaling, independent of the mitochondrial pathway [58]. In type II cells, such as hepatocytes and pancreatic β-cells, lower amounts of active caspase-8 are produced at the DISC level, making additional signaling necessary for apoptosis to occur. This additional signaling involves the mitochondrial pathway [57].

### 3.2. Non-Canonical TRAIL Signaling Pathway

**Figure 2:**
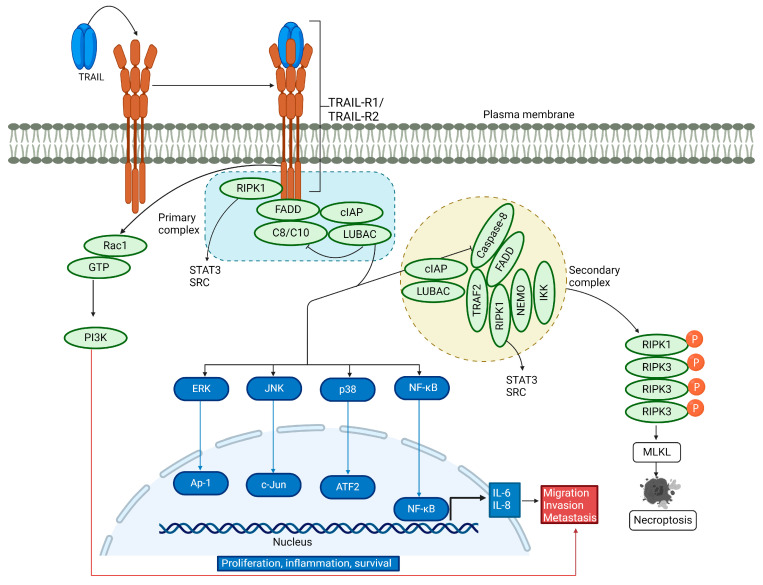
Non-canonical TRAIL signaling pathway. Upon binding of TRAIL to its receptors, a secondary cytosolic complex is formed, consisting of FADD and caspase-8, which interact with RIPK1, tumor necrosis factor (TNF) receptor-associated factor 2 (TRAF2), and nuclear factor kappa light chain enhancer of activated B cells (NF-κB) essential modifier (NEMO). TRAF2 recruits cellular inhibitor of apoptosis proteins 1/2 (cIAP1/2) and the linear ubiquitin chain assembly complex (LUBAC), which attaches linear polyubiquitin chains to RIPK1. These complexes activate NF-κB, p38 MAPK, JNK, and ERK pathways. LUBAC is present in both complexes and aids in recruiting the IKK complex, leading to NF-κB activation. RIPK1 also triggers the activation of tyrosine-protein kinase SRC and STAT3, promoting cell invasion and migration. For a description of necroptosis, see the text.

In addition to inducing apoptosis, TRAIL is involved in non-canonical signaling (Figure 2). It acts as a pro-survival, pro-inflammatory, and proliferative molecule, increasing cell migration and invasion [59,60,61,62,63,64,65]. TRAIL is known to induce nuclear factor kappa B (NF-κB) activation and promotes a pro-inflammatory response [34,66,67,68,69]. Reports have shown that receptor-interacting serine/threonine protein kinase 1 (RIPK1) is implicated in TRAIL-mediated NF-κB induction through the activation of the inhibitor of the κB (IκB) kinase complex (IKK complex), which phosphorylates IκB, followed by its polyubiquitination and subsequent degradation by the proteasome. Consequently, the released NF-κB can translocate into the nucleus and activate its target antiapoptotic genes [70,71,72,73,74]. TRAIL-mediated activation of NF-κB, which leads to cell proliferation and survival, has been demonstrated by inhibiting NF-κB in cells lacking an inhibitor of nuclear factor-κB (IκB) kinase γ (IKKγ) and receptor-interacting protein kinase 1 (RIPK1) or in cells overexpressing dominant-negative IκBα [75]. Conversely, another report showed that TRAIL can induce cell migration and invasion without affecting cell proliferation, mediated by NF-κB [64].

In the non-canonical signaling pathway, TRAIL binding to its cognate receptor leads to the association of a secondary DISC complex, which is proposed to activate not only NF-κB but also the JUN N-terminal kinase (JNK) and p38 mitogen-activated protein kinase (p38 MAPK) pathways [59,76]. TRAIL-activated kinase pathways initiate a gene-activating signaling cascade that results in cytokine synthesis such as interleukin-8 and monocyte chemoattractant protein-1, leading to macrophage migration [76]. Additionally, TRAIL promotes cell proliferation by engaging TRAIL-R2 in the extracellular signal-regulated kinase (ERK) 1 and 2 pathway, even in the absence of caspase-8 [77]. Another study showed that TRAIL significantly stimulates the extracellular signal-regulated kinase 1/2 via binding to TRAIL-R2, thus participating in the non-apoptotic pathway [78]. Recent experiments have revealed that TRAIL-R2 can translocate to the nucleus and interact with proteins of the microprocessor components, thereby preventing the maturation of the microRNA let-7 and inducing cell proliferation [79,80]. TRAIL-mediated activation of RIPK1 promotes pro-survival through protein kinase B (AKT) signaling and cell migration by triggering the proto-oncogene tyrosine-protein kinase Src and the signal transducer and activator of transcription 3 (STAT3) pathways [81]. In a distinct signaling mechanism, a ten-amino-acid membrane-proximal stretch of TRAIL-R2 activates Ras-related C3 botulinum toxin substrate 1 (Rac1), independently of complex I and II formation, leading to cell invasion and migration via the PI3K pathway [61,82]. Hence, TRAIL-mediated activation of the non-canonical pathway induces the activation of multiple kinase signaling pathways, leading to survival, proliferation, migration, and invasion in a cell-type-dependent manner.

### 3.3. TRAIL-Induced Necroptosis

Under specific cellular conditions, such as acidic pH [83,84], the depletion of cellular inhibitor of apoptosis (cIAP) proteins [85], or the reduction of TNF receptor-associated factor 2 (TRAF2) [86,87], the TRAIL-induced cell death pathway may shift towards necroptosis. This pathway requires RIPK1 [88] and recruits RIPK3, which is subsequently phosphorylated and then phosphorylates and activates the pseudokinase mixed lineage kinase domain-like protein (MLKL) [89,90,91,92,93,94,95,96,97]. Upon activation, MLKL forms oligomers that localize to the plasma membrane, compromising its integrity and the ability to maintain ionic homeostasis. As a result, ion influx occurs, leading to cell swelling, plasma membrane rupture, and the subsequent release of intracellular contents [98,99,100] (Figure 2).

## 4. TRAIL’s Role in the Innate and Adaptive Immune Systems

TRAIL plays several crucial roles in maintaining immune system homeostasis, including immunoregulatory, immunosuppressive, and immune-effector functions, as well as facilitating the transition from innate to adaptive immunity [101]. While studies have shown that TRAIL is involved in central and peripheral tolerance, its role in central tolerance remains controversial.

Initial investigations into TRAIL signaling in the negative selection of human and mouse thymocytes indicated that TRAIL is not essential for central tolerance and does not play a significant role in thymocyte negative selection [102]. However, other studies revealed that TRAIL deficiency in knockout mice led to dysfunctional thymocyte apoptosis and impaired negative selection of autoreactive thymocytes [103]. In contrast, other studies using TRAIL knockout mice and anti-TRAIL antibodies suggested that TRAIL is not critical for thymocyte negative selection [104]. Additionally, TRAIL-R-deficient mice showed no differences in thymocyte negative selection compared to their wild-type littermates [105]. Despite these findings, Corazza and colleagues proposed that TRAIL may indirectly influence thymocyte apoptosis via a mitochondria-dependent pathway, implicating TRAIL in thymic negative selection [106].

TRAIL is also crucial in maintaining peripheral immune tolerance through multiple pathways. One such mechanism involves the removal of activated immune cells via activation-induced cell death (AICD) [107], which helps prevent the potential induction of an autoimmune response. TRAIL has been shown to induce apoptosis in T cells following IL-2 stimulation [108] and is essential for AICD in human peripheral blood mononuclear cells [107]. Another study found that TRAIL is expressed in T helper 2 (Th2) cells, where the membrane-bound form can mediate the apoptosis of CD4^+^ Th1 cells, indicating TRAIL’s significant role in T helper cell differentiation [109,110]. Moreover, TRAIL can induce apoptosis in antigen-activated CD8^+^ T cells that proliferate in the absence of CD4^+^ T cells [111]. Additionally, engaging TRAIL-R with TRAIL and stimulation with anti-CD3/CD28 can block T cell proliferation, silencing T cell activation without significant apoptosis [112]. TRAIL’s role in peripheral tolerance is further demonstrated by its ability to promote the expansion of T regulatory cells (Tregs), which are involved in suppressing autoimmunity [113]. The critical importance of Tregs in the development of T1D and other autoimmune diseases is underscored by mutations in the forkhead box P3 (FOXP3) gene [114,115,116]. Additionally, TRAIL can induce cell cycle arrest in CD8^+^ cells by reducing cyclin-B1 levels, thereby blocking the cell cycle in the G2/M phase [117,118].

Numerous studies have demonstrated that TRAIL plays an anti-inflammatory role. In human patients with cardiovascular disease, lower levels of soluble TRAIL in plasma are associated with increased mortality [119]. In vivo experiments with apoE-deficient mice treated with recombinant TRAIL showed reduced atherosclerotic plaque formation and increased macrophage apoptosis [120]. Double knockout mice lacking both apoE and TRAIL exhibited greater macrophage infiltration and larger atherosclerotic plaques compared to apoE-deficient mice alone [121]. A recent study highlighted the critical role of TRAIL-expressing monocytes/macrophages in protecting against atherosclerosis, evidenced by a higher number of inflammatory macrophages in TRAIL-knockout mice [16].

## 5. Pathology of T1D

Inflammatory susceptibility [122], genetic predispositions, and environmental and infectious agents [123] are considered to contribute to the onset, development, and progression of T1D. Autoantibodies targeting insulin, 65 kDa glutamic acid decarboxylase (GAD65), insulinoma-associated protein 2 (IA-2), and zinc transporter 8 (ZNT8) [124] are associated with T1D. Although these autoantibodies are not regarded as directly pathogenic, they can serve as biomarkers for monitoring the progression of T1D before disease onset [125].

The genetic component plays a significant role in the development of T1D. Genome-wide association studies and candidate gene studies have identified genetic loci linked to varying degrees of risk for developing T1D [126,127]. The strongest association is with the human leukocyte antigen (HLA) region on chromosome 6p21, particularly HLA-DR and HLA-DQ [128]. The second strongest association is with the insulin gene (INS) variable number of tandem repeats (VNTR) minisatellite, where allelic variation affects insulin mRNA levels [129]. Significant associations with T1D have also been found with lymphoid tyrosine phosphatase (LYP) [130], where gain-of-function mutations suppress T-cell receptor (TCR) signaling [131]. Additionally, polymorphisms in the cytotoxic T-lymphocyte-associated protein (CTLA-4) [132] and interleukin-2 receptor subunit alpha (IL2RA, CD25) [133] genes are linked to T1D. Other associated regions include protein tyrosine phosphatase non-receptor type 2 (PTPN2) [134]; ubiquitin-associated and SH3 domain-containing protein A (UBASH3A) [135]; interferon-induced helicase (IFIH1) [136]; and basic leucine zipper transcription factor 2 (BACH2) [137]. Many of these genes regulate immune cell function, while others directly influence β-cell function in response to inflammation [138].

Studies have shown that various environmental factors significantly impact the development of T1D. One such factor is enteroviruses (e.g., coxsackieviruses), which are suggested to initiate or exacerbate islet inflammation in genetically predisposed individuals [139,140]. Other research indicates that the interaction between the innate immune system and the gut microbiota plays a crucial role in regulating β-cell autoimmunity. Specific alterations in the intestinal microbiome may help prevent islet inflammation and β-cell loss [141,142].

**Figure 3:**
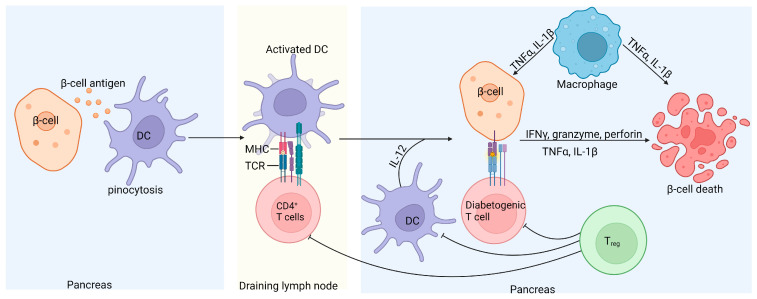
Schematic representation of islet inflammation in T1D. Self-antigens released by damaged β-cells recruit antigen-presenting dendritic cells, which take up the immune complexes, migrate to the pancreatic draining lymph nodes, and activate pathogenic islet antigen-specific T cells. The activated diabetogenic T cells return to the pancreas, where CD8^+^ T cells induce apoptosis of the β-cells by secreting granzyme and perforin or through CD95L-mediated killing. Activated CD4^+^ T cells contribute to β-cell destruction by releasing proinflammatory cytokines. Treg cells can inhibit diabetogenic T cells and consequently prevent β-cell damage through the secretion of IL-10 and transforming growth factor (TGFβ); however, in T1D, Treg cells are defective [146].

Although T1D is a multifactorial disease, it is primarily considered a T-cell-mediated autoimmune disorder resulting from defects in central and peripheral tolerance and self-antigen processing [8]. It is well documented that islet-specific autoreactive CD4^+^, CD8^+^, and B cells contribute to the loss of insulin-producing pancreatic β-cells (Figure 3) [101,143,144,145].

Studies involving non-obese diabetic (NOD) mice have shown that CD8^+^ T cells can initiate and induce T1D by directly targeting and killing pancreatic β-cells [147]. Other studies have demonstrated that CD4^+^ T cells are involved in both the initiation and progression of T1D through various mechanisms. Research on major histocompatibility complex (MHC) class II-restricted CD4^+^ T cells in NOD mice deficient in MHC class II molecules showed that while initial pancreatic infiltration occurs, the progression of insulitis is halted [148]. Conversely, β-cells express MHC class II molecules and can stimulate CD4^+^ T cell proliferation, allowing these effector cells to induce pathology by directly interacting with pancreatic β-cells [149]. Pancreatic CD4^+^ T cells induce β-cell apoptosis through the secretion of inflammatory cytokines such as interferon-gamma (IFN-γ) and tumor necrosis factor-alpha (TNF-α) [150,151]. Additionally, Tregs, characterized by the expression of the transcription factor FOXP3, are believed to be involved in T1D pathogenesis. Tregs’ primary function is to suppress the activation of CD4^+^ and CD8^+^ effector T cells and other immune effectors [8]. Tregs express the CD25 receptor and regulate self-tolerance by binding IL-2, thus depleting this cytokine necessary for effector T cell expansion [152]. Reports have highlighted the significance of the relationship between IL-2 and CD25 receptors in T1D. In NOD mice, reduced IL-2 secretion by effector T cells correlates with intra-islet Treg cell apoptosis and dysfunction [153]. In humans, polymorphisms in the IL2R haplotype have been associated with reduced IL-2 signaling in CD4^+^ T cells exposed to antigens, downregulating the FOXP3 gene and resulting in the limited suppression of autologous effector T cells [154]. These investigative studies indicate that T1D is primarily an autoimmune disease, driven by autoreactive T cells that target insulin-producing pancreatic β-cells.

## 6. TRAIL Implications in T1D

Many studies have shown that TRAIL is involved in transmitting both apoptotic and non-apoptotic signals across various cell types [155,156,157,158]. Research on TRAIL in the context of T1D reveals a complex role, demonstrating both protective and pro-apoptotic effects [159]. The pro-apoptotic activity of TRAIL has been extensively studied in vitro. For instance, TRAIL has been shown to induce apoptosis in pancreatic β-cell lines more effectively compared to other pathways, such as FasL, TNF-α, lymphotoxin (LT) α1β2, LIGHT, and IFN-γ [160]. However, freshly isolated islet cells exhibit resistance to TRAIL-induced cytotoxicity [160]. Additionally, studies on isolated primate islets have shown that blocking TRAIL through its decoy receptors increases islet cell viability [161]. The mechanism of β-cell loss induced by TRAIL involves two critical apoptotic components, FADD and nuclear factor κB (NF-κB), which work synergistically [160,162]. Moreover, TRAIL-mediated cytotoxicity and apoptosis are reduced in human β-cell lines and primary islet cells when these cells overexpress anti-apoptotic proteins such as BCL-2 and XIAP [162].

In contrast to TRAIL’s negative pro-apoptotic role in T1D, its protective features have been demonstrated in various animal models. The most commonly used mouse models for studying T1D are the streptozotocin (STZ)-induced diabetes model and NOD mice. In STZ-induced diabetic mice, diabetes is chemically induced using STZ, a compound that selectively destroys pancreatic beta cells [163], leading to insulin deficiency. STZ functions by causing DNA damage in beta cells, which results in their destruction and a rapid onset of diabetes [164]. While this model effectively mimics the hyperglycemia observed in diabetes [165], it does not replicate the autoimmune component seen in human T1D. In contrast, diabetes in NOD mice develops spontaneously due to a genetic predisposition to autoimmunity [166]. NOD mice develop T1D as a result of an autoimmune attack on pancreatic beta cells, closely resembling the pathology of human T1D [167]. In these mice, T cells infiltrate the pancreatic islets (a condition known as insulitis), gradually leading to beta cell destruction [168].

STZ-induced diabetes in TRAIL-deficient mice developed diabetes earlier than wild-type mice, and their pancreatic islets showed severe inflammation [11]. Similarly, blocking TRAIL by injecting its soluble receptor DR5 into cyclophosphamide (CY)-treated NOD mice, to accelerate diabetes onset, resulted in disease characteristics similar to those seen in TRAIL-deficient mice treated with STZ. [11]. In STZ-induced diabetic rats transplanted with TRAIL-transduced islets, blood glucose levels normalized for up to 60 days, and insulitis was reduced compared to rats grafted with mock-infected islets [169]. Additionally, injecting recombinant TRAIL into STZ-induced diabetic mice reduced pancreatic islet inflammation and lowered blood glucose levels [170]. Recombinant TRAIL was also shown to upregulate the suppressor of cytokine signaling 1 (SOCS-1) and downregulate cytokines involved in systemic (TNF-α and OPG) and pancreatic (VCAM-1) inflammation [170]. SOCS-1 is a molecule demonstrated to inhibit the signal transduction pathways triggered by inflammatory cytokines [171]. Notably, transgenic NOD mice expressing SOCS-1 exhibit a lower incidence of diabetes due to inhibited IFN-γ response, which is crucial in T1D pathogenesis [172]. Moreover, adenoviral delivery of SOCS-1 to pancreatic islet grafts before transplantation inhibits the apoptotic pathway and extends graft survival [173]. Thus, SOCS-1 expression in pancreatic islets protects β-cells by blocking pro-apoptotic signaling and enhancing cell survival. Additionally, TRAIL has been shown to protect against T1D by halting the proliferation of diabetogenic T cells rather than increasing activation-induced cell death [174]. This protection is associated with lower IL-2 levels and the inhibition of cell cycle progression. TRAIL promotes the expression of the cyclin-dependent kinase inhibitor p27^kip1^, which blocks diabetogenic T cells at the early G1 phase of the cell cycle and induces a state of anergy [174]. Furthermore, adenoviral delivery of TRAIL in NOD mice increased tissue inhibitor of metalloproteinase-1 (TIMP-1) expression, which protected against T1D and inhibited cytokine-induced β-cell apoptosis [10]. Elevated TIMP-1 also led to the downregulation of matrix metalloproteinase (MMP) activity, which was associated with reduced diabetogenic T cell transmigration into pancreatic islets and an anti-inflammatory effect [10]. TIMP-1 transgenic mice treated with low doses of STZ exhibited lower insulitis, increased β-cell proliferation, normal blood glucose levels, and improved survival compared to non-transgenic mice [175]. Although various animal models have been used to study the role of TRAIL in T1D, the results consistently demonstrate the protective function of TRAIL. In humans with T1D, significantly reduced circulating TRAIL levels have been observed, with the lowest levels found in patients with ketoacidosis at onset and those requiring high levels of insulin [12]. Additionally, a correlation has been observed between TRAIL levels and metabolic status, with lower circulating TRAIL levels associated with worse metabolic status [176].

## 7. Conclusions

T1D is a highly heterogeneous disorder characterized by a range of contributing factors, including genetic, epigenetic, and environmental influences, as well as immune system dysregulation leading to islet inflammation. Extensive research has shown that CD4^+^, CD8^+^, and B cells play significant roles in the pathogenesis of T1D. TRAIL, known for its roles in tumor suppression and the clearance of certain viral infections, also plays a crucial role in the transition from innate to adaptive immunity. Given that T1D is an autoimmune disease and TRAIL is involved in immune regulation, a connection between TRAIL and T1D is plausible. In vivo studies have demonstrated TRAIL’s ability to prevent both the development and progression of T1D. The protective effects of TRAIL have been observed in both animal models and in vitro studies. The inhibition of TRAIL activity or TRAIL knockout has been shown to worsen T1D. Systemic TRAIL administration in mice has been shown to alleviate pancreatic islet inflammation. Furthermore, T1D patients exhibit lower circulating levels of TRAIL at disease onset and during later stages of the disease, with TRAIL levels increasing following the initiation of antidiabetic treatment. Overall, these research studies demonstrate that TRAIL has a protective effect by reducing the proliferation of diabetogenic T cells and pancreatic islet inflammation. Based on human clinical data, further research is needed on the modulation of serum TRAIL levels before it can be considered a candidate molecule for T1D clinical trials. However, to fully understand the relationship between TRAIL and T1D, as well as its protective role, and to effectively intervene in the progression of islet inflammation and beta cell destruction, further studies—both in vitro and in vivo—are still necessary to gain deeper insights into the mechanism of action of this intriguing molecule.

## Data Availability

The original contributions presented in the study are included in the article, further inquiries can be directed to the corresponding author.
